# Reactive Oxygen Species and Nrf2: Functional and Transcriptional Regulators of Hematopoiesis

**DOI:** 10.1155/2019/5153268

**Published:** 2019-11-18

**Authors:** Linping Hu, Yawen Zhang, Weimin Miao, Tao Cheng

**Affiliations:** ^1^State Key Laboratory of Experimental Hematology, China; ^2^National Clinical Research Center for Blood Diseases, Institute of Hematology & Blood Diseases Hospital, China; ^3^Chinese Academy of Medical Sciences & Peking Union Medical College, Tianjin 300020, China; ^4^Center for Stem Cell Medicine, Chinese Academy of Medical Sciences, Tianjin, China; ^5^Department of Stem Cell & Regenerative Medicine, Peking Union Medical College, Tianjin, China

## Abstract

Hematopoietic stem cells (HSCs) are characterized by self-renewal and multilineage differentiation potentials. Although they play a central role in hematopoietic homeostasis and bone marrow (BM) transplantation, they are affected by multiple environmental factors in the BM. Here, we review the effects of reactive oxygen species (ROS) and Nrf2 on HSC function and BM transplantation. HSCs reside in the hypoxic microenvironment of BM, and ROS play an important role in HSPC regulation. Recently, an extraphysiologic oxygen shock/stress phenomenon was identified in human cord blood HSCs collected under ambient air conditions. Moreover, Nrf2 has been recently recognized as a master transcriptional factor that regulates multiple antioxidant enzymes. Since several years, the role of Nrf2 in hematopoiesis has been extensively studied, which has functional similarities of cellular oxygen sensor hypoxia-inducible factor-1 as transcriptional factors. Increasing evidence has revealed that abnormally elevated ROS production due to factors such as genetic defects, aging, and ionizing radiation unexceptionally resulted in lethal impairment of HSC function and hematopoiesis. Both experimental and clinical studies have identified elevated ROS levels as a major culprit of ineffective BM transplantation. Lastly, we discuss the possibility of using small molecule antioxidants, such as N-acetyl cysteine, resveratrol, and curcumin, to augment HSC function and improve the therapeutic efficacy of BM transplantation. Further research on the function of ROS levels and improving the efficacy of BM transplantation may have a great potential for broad clinical applications of HSCs.

## 1. Introduction to the Hematopoietic System

Bone marrow (BM) transplantation has achieved great success in clinical practice and has saved numerous lives. Nevertheless, further improvements in its therapeutic efficacy are warranted. Various lineages of blood cells have been derived from common hematopoietic stem cells (HSCs) [1]. Hematopoiesis occurs in a hierarchical manner, with HSCs at the top as the cells of origin. They are able to self-renew and differentiate into various lineages of peripheral blood cells via hematopoietic progenitor cells [2–4]. HSCs have two basic properties: self-renewal and multipotent differentiation [4, 5]. However, molecular control underlying the stemness of these cells is still unclear and is thus a topic of interest.

HSC function is regulated by both intrinsic and extrinsic factors. Intrinsic factors arise from uniquely expressed signaling pathways in HSCs, whereas extrinsic factors arise from multiple factors in the microenvironment where HSCs reside, e.g., the BM niche [6–9]. Advances in single-cell and molecular technologies have led to a better understanding of the BM niche, both at homeostasis and under acute myeloid leukemia conditions [7, 10]. BM niche represents a three-dimensional space comprising several types of components such as cells, blood vessels, extracellular matrices, cytokines, and adhesion molecules. Mesenchymal stem cells (MSCs), osteolineage cells, bone marrow-derived endothelial cells, chondrocytes, fibroblasts, and pericytes compose most niche cells, which interact with HSCs and regulate the function of HSCs [10]. Reactive oxygen species (ROS) represent another metabolic niche factor that has attracted increasing attention [11, 12]. Moreover, Nrf2 has been recently recognized as a master transcriptional factor that regulates multiple antioxidant enzymes. Thus, in this article, we have summarized advancements in the assessment of the effects of ROS and Nrf2 on HSC function and BM transplantation.

## 2. Sources of ROS and Cellular Redox Homeostasis

**Figure 1:**
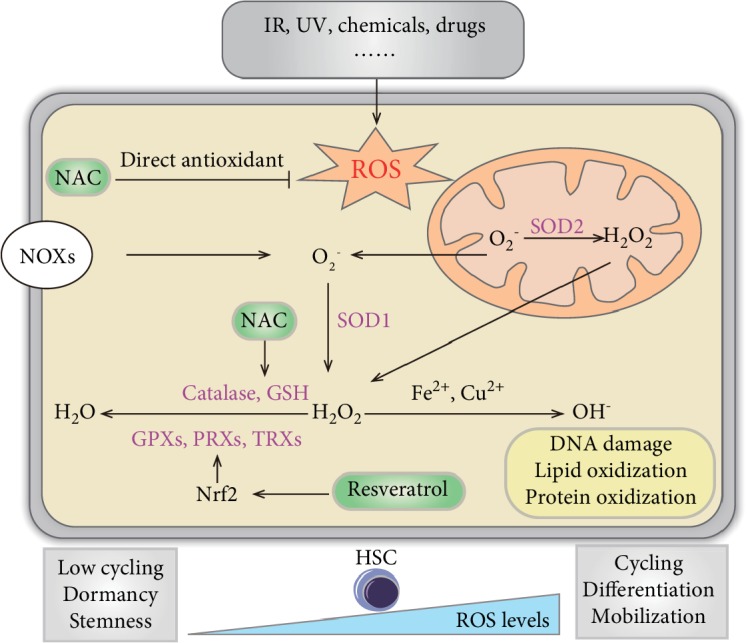
Sources of ROS and regulation cellular redox homeostasis in hematopoiesis. Ionizing radiation (IR), ultraviolet (UV), chemical drugs, etc. result in exogenous elevated ROS levels. Accumulated O2^−^ molecules by mitochondria and membrane NADPH oxidase (NOX) in cells are first converted by superoxide dismutase (SOD) into H_2_O_2_. H_2_O_2_ quickly converts into harmless water (H_2_O) in the presence of catalase, glutathione (GSH), glutathione peroxidase (GPX), peroxiredoxin (PRX), and thioredoxin (TRX). If cells are insufficient to detoxify H_2_O_2_, the remaining H_2_O_2_ is converted into even more toxic OH^−^ ions. OH^−^ ions can lead to destruction of cellular macromolecules including proteins, nuclear acids, and lipids. N-acetyl cysteine (NAC) and resveratrol are two small molecule antioxidants which reduce ROS levels. In hematopoiesis, dormant, quiescent HSCs are characterized by low ROS levels. Elevated ROS levels in HSCs enhance cycling and promote differentiation and mobilization.

Endogenous ROS are primarily derived from oxidative metabolism in the mitochondria, physiological metabolism processes, and inflammatory reactions. The examples of ROS include superoxide anion (O_2_^−^), hydrogen peroxide (H_2_O_2_), and hydroxyl ion (OH^−^). Several hypotheses exist regarding the sources of ROS; however, mitochondria and membrane NADPH oxidase (NOX) are the two most recognized sources [13, 14]. During cell proliferation, numerous biological macromolecules are involved in signal transduction and energy metabolism; ROS are formed as byproducts of these two processes. Current studies have revealed that ROS are not always harmful to cells; by contrast, ROS can serve as important signaling molecules [15–17]. ROS levels fluctuate during different cell cycle states of HSCs, affecting their motility, proliferation, differentiation, and repopulation potential. Also, elevated ROS levels in HSCs and MSCs promote HSC migration and mobilization [18]. Recently, Lapidot's group revealed that the oscillatory ROS levels in hematopoietic stem and progenitor cells (HSPCs) were driven by light and dark signals through different mechanisms. Those resulted in BM HSPC differentiation and replenishment with mature blood cells during the day and replenishment of the BM pool of stem and progenitor cells at night [19]. However, when ROS levels become abnormally high, HSCs may initiate a protective mechanism to shut down self-renewal functions. High ROS levels are known to cause cellular DNA damage and cell cycle arrest. Subsequently, DNA damage repair is initiated in affected cells. If the damage is successfully repaired, cells may continue to proliferate and differentiate. However, if the damage is too severe to be repaired, cells may undergo senescence or apoptosis [20]. Over the course of evolution, a complex defensive network has arisen to scavenge ROS to maintain redox balance in cells (Figure 1). Briefly, accumulated O_2_^−^ molecules in cells are first converted by superoxide dismutase (SOD) into H_2_O_2_. As H_2_O_2_ is toxic to cells, it is quickly converted into harmless water (H_2_O) in the presence of catalase or glutathione peroxidase (GPX1). However, if cells contain insufficient catalase and GPX1 to detoxify H_2_O_2_, the remaining H_2_O_2_ is converted into even more toxic OH^−^ ions. OH^−^ ions can lead to destruction of cellular macromolecules including proteins, nuclear acids, and lipids [21]. Therefore, ROS detoxification requires a coordinated group of redox modulating enzymes. SOD appears to be a double-sided sword. On the one hand, it is a well-known antioxidant; on the other hand, it is a potent prooxidant, depending on the presence of other antioxidant enzymes such as catalase and GPX1 in cells. Nrf2 was recently discovered as a global regulator of cellular antioxidant defense [22]. Till date, a variety of ROS-detoxifying enzymes have been found to be regulated by the Nrf2/Keap1 pathway as downstream effectors, including SOD, catalase, GPX1, and thioredoxin.

## 3. Role of Hypoxic Environment in Maintaining Stemness of HSCs

It is generally believed that HSCs reside in the hypoxic microenvironment of BM. The oxygen tension of BM was quite low (<32 mm Hg) and heterogenous measured by two-photon phosphorescence lifetime microscopy. The highest oxygen tension in the BM was found next to the endosteum and arteries, while the lowest tension was next to the sinusoids [23]. However, the ROS levels of HSCs which resided in endosteal and arterial areas with high oxygen tension were low, while the ROS levels of HSCs which resided in sinusoids were high unless they were found next to a megakaryocyte who maintained them at low ROS state. The aforementioned phenomenon might be accounted for different blood vessel permeability (less in endosteal and arterial, more in sinusoids) and signals provided by the microenvironment [24]. Manipulation of BM vascular permeability shifted ROS levels in HSCs. Circulating HSCs or HSCs exposed to blood plasma were shown to have the highest ROS levels, increased ability of migration and differentiation, and decreased long-term repopulation potential [24]. Interestingly, when BM or cord blood (CB) is collected or treated in ambient air, HSCs quickly differentiate and significantly decrease in number. The phenomenon was recently reported by Mantel's group, who termed it extra physiologic oxygen shock/stress (EPHOSS) [25]. EPHOSS appears to be associated with elevated ROS. It is mediated by the p53-cyclophilin-mitochondrial permeability transition pore (MPTP) signal axis, which involves hypoxia-inducible factor-1*α* (HIF-1*α*) and *miR210*. To avoid EPHOSS, low oxygen tension (3%) is recommended for HSC collection and handling. An alternative strategy is to collect HSCs under normal oxygen tension but to pretreat the collection tube with cyclosporine A (CSA). The compound CSA, which works on the MPTP axis, can be used to inhibit ROS production and thus improve CB-HSC collection [25, 26]. Moreover, under AML conditions, increased BM vascular permeability resulted in reduced BM oxygen tension, while residual HSCs showed higher levels of ROS and reduced stemness and were mobilized to the periphery [27]. Additionally, it was confirmed that AML cells remodeled endosteal regions and have elevated ROS levels through the proinflammatory and vascular endothelial growth factor signaling pathway [28]. The numbers of healthy hematopoietic cells transmigrating across endothelial cells were increased in leukemic mice and may be associated with the elevated ROS levels [28]. In summary, the ROS levels of HSC were affected by various factors, including location, alternations of vessel permeability, or whether it is under stress.

Moreover, ROS play an important role in HSC regulation. On the one hand, low ROS levels are essential for quiescent HSCs to maintain their stemness; on the other hand, a physiological level of ROS is needed to promote HSC proliferation and differentiation [29]. However, abnormally elevated ROS can result in DNA damage of the CD34^+^ hematopoietic fraction, reduced colony formation, and impaired proliferative capacity [30]. Recently, a novel mechanism of HSPC regulation was verified. A signaling network of p190-B RhoGAP-ROS-TGF-*β*-p38^MAPK^ balances HSPC self-renewal and differentiation [31]. This finding further confirms that ROS play an important role in HSC regulation.

## 4. Role of Nrf2 in Redox Regulation of HSCs and Microenvironment

Nrf2 is well established as a master transcriptional factor regulating various antioxidant enzymes. The role of Nrf2 in hematopoiesis has been extensively studied in recent years. ROS levels are abnormally increased in the liver and kidney cells of *Nrf2* null mice, suggesting that Nrf2 is essential for metabolizing ROS [32]. Kato et al. studied the relationship between radiation sensitivity and *NRF2*-regulated gene expression profiles using CB-HSCs. They performed X-ray irradiation of CD34^+^ human CB-HSCs and then examined the downstream antioxidant genes regulated by *NRF2*. The results showed that after HSCs were irradiated at a dose of 2 Gy, the number of colony-forming cells (CFCs) decreased significantly to 20% of that in the control group. Additionally, multiple antioxidant genes regulated by *NRF2*—such as NADPH dehydrogenase quinone 1 (NQO1), glutathione reductase (GSR), thioredoxin reductase 1 (TXNRD1), heme oxygenase 1 (HO-1), and ferritin heavy chain 1 (FTH1)—were upregulated after irradiation. Although the research is preliminary, the data revealed a critical role of *NRF2* in defensive reactions against ionizing radiation (IR) [33]. Studies using *Nrf2* knockout mice have shown that *Nrf2*^−/−^ HSCs are severely defective and more sensitive to oxidative stress. Surprisingly, ROS levels of *Nrf2*^−/−^ HSCs are at basal levels and the defects of HSCs cannot be restored by treatment with N-acetyl cysteine (NAC). This suggests that induced ROS is not the main cause of HSC defect. However, granulocyte colony-stimulating factor (G-CSF) treatment could rescue HSC function after exogenous oxidative stress despite causing elevated ROS levels which indicates involvement of a cytokine defect in *Nrf2*^−/−^ HSC. High ROS is actually harmful to cells, but in some cases, normal ROS levels cannot represent the undisturbed HSC function; new markers and strategies are needed to measure oxidative stress and cellular damage [34]. In a separate study of *Nrf2*-deficient mice, significant expansion of HSPCs was found to occur at the expense of HSC quiescence and self-renewal. Since the phenotype of *Nrf2*-deficient HSCs is somewhat similar to that of forkhead O- (FOXO-) deficient HSCs, the author speculated that Nrf2 functions in parallel to FOXO proteins as a downstream target of the PI3K-Akt pathway and that this mechanism warrants further investigation [35]. Kim et al. [36] demonstrated that activation of Nrf2 signaling, either by genetic deletion of *Keap1* or by treatment with the small molecule activator 2-trifluoromethyl-2′-methoxychalone (TMC), augments HSPC function and attenuates IR-induced BM suppression and mortality. TMC administration results in activation of Notch signaling in HSPCs and leads to enhanced HSPC expansion [36, 37]. Recently, Murakami et al. [38] demonstrated that Nrf2 drives cell cycle entry and differentiation of LT-HSCs at the expense of their quiescence and maintenance through Keap1. Moreover, the Nrf2 pathway is involved in mediating abnormal hematopoiesis (such as benzene-induced hematotoxicity) in response to oxidative stress [39]. In summary, Nrf2 function vitally in redox regulation of HSCs.

In addition to HSCs, Nrf2 also plays a global role in regulating oxidative stress in niche cells such as MSC, megakaryocytes, Schwann cells, and T cells. Nrf2 known down in MSCs resulted in elevated ROS levels, reduced target antioxidant gene expression, and depressed cell survival. Conversely, reexpression of Nrf2 rescued the increased oxidative stress and restored the *in vitro* and *in vivo* viability of MSCs [40]. Nrf2 was also proved to maintain stem cell properties and the osteogenesis process in MSCs [41]. Moreover, by competing with Nrf2, NF-E2 p45 regulated cytoprotective genes and promoted elevated ROS levels to enhance megakaryocytic maturation [42]. Also, *Nrf2*^−/−^ mice showed impaired functional recovery after peripheral nerve injury [43]. Slight oxidative stress activated Nrf2, enhanced the expression of antioxidant enzymes such as catalase and HO-1, promoted neurons to generate glutathione (GSH), and thus ultimately protected sensory neurons and Schwann cells from oxidative damage [44]. A critical role of Nrf2 in T cell function has been revealed by Brink's group. Expression and nuclear translocation of Nrf2 were significantly increased after T cell activation *in vitro* and CD4^+^ donor-derived T cells. *Nrf2*^−/−^ donor-derived T cells induced less acute graft-versus-host disease through increased Helios^+^ donor regulatory T cells. In detail, Nrf2 reduced CD25 expression and moderated IL-2 signaling resulting in decreasing Helios and Foxp3 expression [45]. In conclusion, Nrf2 protects niche cells from oxidative stress and creates a favorable microenvironment for HSCs.

As a well-established global regulator of redox homeostasis, Nrf2 plays an essential role in other stem cell types, including embryonic stem cells (ESCs), MSCs, and cancer stem cells (CSCs). For example, Nrf2 is involved in the control of self-renewal properties and pluripotency in human ESCs [46] and is essential for maintaining quiescence and differentiation in adult stem cells including *Drosophila* intestinal stem cells [47], human airway basal stem cells [48], and human MSCs [40]. Nrf2 also contributes to maintenance of quiescence, survival, and stress resistance in CSCs [49, 50].

## 5. Genetic Defects Unveiling the Signaling Pathways for Redox Regulation in HSCs

Studies using genetically deficient mouse models have shed light on the signaling pathways that mediate ROS-induced impairment in HSCs [13, 21]. For example, ATM mutation has been shown to lead to ataxia telangiectasia, an autosomal recessive genetic disorder in humans. Aged *Atm*^−/−^ mice display progressive BM failure due to severe defects in HSC function. These HSC defects are associated with high levels of ROS, since treatment with the antioxidant NAC significantly improves HSC reconstitution capacity in *Atm*^−/−^ mice [51]. Mechanistic studies have further revealed that high ROS levels in *Atm*^−/−^ mice activate the p38MAPK pathway, contributing to HSC exhaustion. Treatment with a p38 inhibitor restores ROS-induced defects in HSC reconstitution ability and in the maintenance of HSC quiescence [52].

The FoxO family of transcription factors plays active roles in regulating genes involved in cell growth, proliferation, differentiation, and metabolism. The family comprises four members, namely, FoxO1, FoxO3, FoxO4, and FoxO6, present in mammalian cells. Studies [53] have shown that FoxO transcription factors are essential for controlling redox homeostasis of HSCs. Gilliland's group created conditional gene deletions for *Foxo1*, *Foxo3a*, and *Foxo4*. In *FoxO* triple-deletion mice, the number of hematopoietic LSK (Lin-Scal-1+C-Kit+) cells was markedly decreased. Moreover, HSCs from *FoxO*-deficient mice had a poor long-term repopulating capacity due to increased differentiation and apoptosis [53]. Notably, the HSC defects were associated with high ROS levels, which resulted in DNA damage [54], and the FoxO HSC phenotype could be reversed by treatment with NAC [55, 56]. Although FoxO family members have both distinct and overlapping functions, a single *FoxO3a* deletion can result in a similar defect in HSC long-term repopulating activities associated with high ROS levels. High ROS levels have been shown to lead to phosphorylation of p38MAPK in *FoxO3a*-deficient cells, which activates the cellular senescence-related genes *p16^Ink4a^* and *p19^Arf^* and consequently results in loss of HSC function. Additionally, the HSC defects could be repaired by treatment with p38 inhibitors or the antioxidant NAC [57]. Although several upstream signaling pathways may be involved, PI3K/Akt is mostly recognized for its negative regulation of FoxO transcriptional factors [58]. Furthermore, the *microRNA-212/132* cluster is known to regulate expression of *FoxO3*, and its overexpression or knockout can lead to hematopoietic defects [59].

Fanconi anemia (FA) is a genetic disorder characterized by progressive BM failure, congenital defects, and predisposition to cancer. FA is caused by a defect in any of the 16 FA genes (FANCA-Q), all of which are involved in the DNA repair pathway. FANCD2 is downstream of a large nuclear complex composed of multiple FA proteins. *Fancd2*-deficient mice have a variety of HSC defects including loss of quiescence, abnormal cell cycle, and compromised function. Li's group reported an oxidative stress-specific interaction between FANCD2 and FOXO3a: a novel pathway in which FANCD2 and FOXO3a colocalize and cooperate was found to be involved in the cellular oxidative stress response [60]. To study the impact of this pathway on hematopoiesis, the authors constructed *Fancd2* and *Foxo3a* double-knockout (DKO) mice. Deletion of both *Fancd2* and *Foxo3a* resulted in an initial expansion followed by progressive reduction of HSPCs. DKO HSCs showed dramatic reduction in hematopoietic reconstitution ability in transplantation experiments. Moreover, Fancd2 was shown to be required for nuclear retention of Foxo3a, *Fancd2* deficiency kept Foxo3a localized in the cytoplasm of HSCs, and reexpression of *Fancd2* rescued Foxo3a nuclear localization [61].

Besides genetic regulation, epigenetic regulation from noncoding RNA is also involved in redox regulation of hematopoiesis. Qian et al. reported that the mammalian imprinted Dlk1-Gtl2 locus plays an important role in sustaining long-term HSC properties [62]. Mechanistic studies have demonstrated that the miRNA megacluster within the Dlk1-Gtl2 locus inhibits the entire PI3K-mTOR pathway, which in turn suppresses mitochondrial biogenesis and reduces ROS production [62].

## 6. Excessive ROS Production in Hematopoietic Tissue during Aging

It has long been observed that HSCs from aging mice have low homing and engraftment efficiencies [63] and poor therapeutic efficacy [64]. Although it is taken for granted that aging compromises HSC function [65], the precise molecular mechanism is yet to be elucidated. Elevated ROS levels have been speculated as one of the causes of HSC defects in aging mice. Meyrelles et al. conducted comparison experiments among the young-, intermediate-, and old-age groups (younger than 2, 12, and 24 months, respectively) of C57BL/6J mice. Their data showed that intracellular ROS levels in HSCs were significantly higher in the old-age group than in the young-age group. Moreover, the ROS sources were different. Mitochondrial and NOX sources were common in all three groups while the CYP450 source was observed in the intermediate- and old-age groups; by contrast, the xanthine oxidase source was only observed in the old-age group. In old-age mice, high levels of ROS were associated with more differentiated HSCs. “Aging” HSCs showed high levels of DNA damage and high rates of senescence and/or apoptosis [66]. Khatri et al. found that, in aged mice, BM stromal cells accumulate more ROS than do HSCs, which considerably reduces cellularity in the BM. Notably, elevated ROS levels compromise the ability of BM stromal cells to support HSC reconstitution in aged mice [67]. Treatment with the antioxidant curcumin has been shown to significantly improve hematopoietic reconstitution in aged mice. In addition, favorable effects of the ginsenoside Rg1 [68] and angelica sinensis polysaccharide (ASP) [69] on HSPCs via suppression of ROS in an induced aging mouse model have been reported. In another study, aged microenvironment was shown to contribute to the aging of HSCs. Aged endothelial cells showed increased ROS levels and promoted myeloid-biased hematopoiesis and HSC functional decline. Young ECs could protect the aged BM microenvironment and rejuvenate the aged hematopoietic system. And young ECs, which have lower ROS levels, may act as a supportive clinical cellular therapy [70]. Taken together, excess production of ROS by HSCs and niche cells during aging accounted for HSC defects. Hence, preventing excessive ROS production could be a feasible method to promote rejuvenation of aged hematopoietic tissue.

## 7. Elevated Cellular ROS Levels Induced by Exogenous Insults

The most studied exogenous insult is IR [68]. IR exposure resulting from nuclear accidents, terrorist attacks, or radiation therapy represents a major medical concern. BM is one of the most susceptible tissues to IR. IR is known to cause acute and long-term BM suppression, and patients with BM impairments after IR exposure may experience severe hematological symptoms or even death. ROS have been confirmed as the major cause of long-term BM suppression after irradiation. High levels of ROS induced by IR greatly impair HSC self-renewal and induce senescence, ultimately leading to HSC exhaustion. Further research has shown that ROS-induced HSC senescence is mediated by redox-dependent activation of the p38^MAPK^-p16^ink4a^ pathway [71]. Recently, low-dose irradiation, which was previously linked to leukemia, was proven to promote persistent oxidative stress and decrease self-renewal of HSCs [72]. Importantly, treatment with the antioxidants resveratrol [73], isorhapontigenin, heyneanol-A [74], metformin [75], chlorophyllin [76], 3,3′-diindolylmethane [77], or methoxytryptamine-*α*-lipoic acid [78] can effectively reduce IR-induced ROS, thereby preventing or mitigating IR-induced hematopoietic dysfunctions.

Besides IR, various environmental toxins can induce cellular ROS production [79, 80]. For example, lead is a heavy metal environmental toxin that can impair various organs and tissues, including the hematopoietic system. Elevated cellular ROS levels are regarded as a major mechanism of lead toxicity. Liu et al. reported that lead-exposed rats have developmental defects in myeloid and lymphoid lineages. Indeed, lead exposure results in senescence/apoptosis and functional loss of HSCs, all of which are associated with mitochondrial defects and increased ROS generation [80]. Treatment with the antioxidant vitamin C can alleviate the functional decrease in lead-exposed HSCs [80]. The adverse effect of iron overload on HSCs offers further evidence. Iron overload due to congenital hemochromatosis or repeated blood transfusions can lead to BM failure and parenchymal organ disorders. Lu et al. [81] studied the effect of iron overload on hematopoiesis in patients with transfusional iron overload. *In vitro* experiments have demonstrated that iron overload results in apoptosis, cell cycle arrest, and compromised function of BM-derived mononuclear cells and umbilical cord-derived MSCs, which are correlated with elevated ROS levels and activated p38MAPK and p53. Importantly, NAC or deferoxamine (DFO) can mitigate iron overload-induced HSC impairments [81]. Using a mouse model, Chai et al. [82] demonstrated that iron overload significantly decreased the proportion and clonogenic function of murine HSPCs. The impairments were associated with elevated ROS levels and could be corrected by treatment with NAC or deferasirox (DFX). These treatments remarkably reduced ROS levels by suppressing NOX4 and p38^MAPK^ and improved hematopoietic reconstitution of HSCs impaired by iron overload after transplantation [82].

## 8. Adverse Effects of ROS on BM Transplantation

Numerous experiments with mouse models have indicated that elevated ROS levels are a major negative factor for BM transplantation. We reported that recipient mice produce high levels of ROS after total body irradiation (TBI) (before transplantation), which can elicit a type of “bystander effect” on transplanted HSCs [83]. The affected HSCs then undergo proliferation-independent exhaustion, which is marked by a dramatic decrease in the expression of the *c-kit* gene and is correlated with elevated ROS levels. Again, administration of NAC can reverse the harmful effects and significantly improve transplant engraftment. Another study [84] by our group showed that the baseline expression levels of two major ROS-metabolizing enzymes, namely, MnSOD and catalase, are very low in HSCs. Injection of MnSOD plasmid liposomes into recipient mice before TBI markedly reduced ROS levels in the BM, which significantly improved HSC transplant engraftment. The authors [84] also successfully constructed a retroviral vector to overexpress MnSOD or catalase in mouse HSCs. Overexpressed catalase had a marked positive effect on long-term reconstitution of transplanted HSCs, and this effect was further augmented after another sublethal irradiation challenge in the transplanted mice. However, overexpressed MnSOD had a less beneficial effect than catalase on the reconstitution of transplanted HSCs.

Clinical investigations have confirmed our finding of the negative effects of ROS on BM transplantation in mouse models [83–85]. Using a prospective paired study, Huang's group found that an elevated ROS level was a major risk factor for “poor graft function (PGF)” after allogeneic BM transplantation. Mechanism studies have further revealed that ROS cause DNA damage, HSC apoptosis (especially CD34+CD38- HSC), HSC exhaustion, and defective CFU formation. Increased levels of p53, p21, caspase-3, and caspase-9 (but not p38) have also been detected in ROS-affected HSCs [86].

## 9. Potential Application of Small Molecule Antioxidants in BM Transplantation

As discussed earlier, mobilized peripheral HSCs showed elevated ROS levels due to the G-CSF treatment [18], exposure to blood plasma elements [24], and EPHOSS [25]. Thus, reducing ROS levels through *ex vivo* small molecule antioxidant treatment, low oxygen tension for HSC collection, and exogenous Nrf2 activation could be feasible methods to improve engraftment efficiency via maintaining stemness of HSC. In addition, *in vivo* reducing ROS levels post transplantation in the BM microenvironment could promote better niche function for an increase in hematopoietic engraftment.

The most widely used small molecule antioxidant in hematopoiesis research is likely NAC, a medication used to treat paracetamol overdose, mucolytics, hemorrhagic cystitis, and obstructive lung disease, among others. It is also sold as a dietary supplement for its antioxidant effects. NAC is a precursor of L-cysteine and reduced GSH which functions as the antioxidant in a direct and indirect way. NAC directly scavenges O_2_^−^, H_2_O_2_, OH^−^, and other free radicals through the -SH thiol group to exert direct antioxidant activity [87]. Also, NAC acts as an indirect antioxidant by increasing the intracellular GSH concentration to reduce redox stress [88] (Figure 1). Numerous studies to date have used NAC to improve the therapeutic effects of BM transplantation. NAC has also been used to assess the impact of ROS on HSCs, restore genetic defects in the redox pathway, overcome the bystander effect caused by IR, and improve BM transplantation efficiencies in immunodeficient mice (NOD/SCID) [51, 55, 81–83]. NOD/SCID mice are commonly used for *in vivo* transplantation studies of human HSCs; however, the engraftment efficiency is unsatisfactory. Our group [85] detected higher levels of ROS in the BM of NOD/SCID mice relative to other normal mouse strains (C57BL/6 and BALB/C). Administration of NAC was found to reduce ROS levels in the BM of NOD/SCID mice. Moreover, NAC treatment significantly improved human HSC engraftment and multilineage hematopoietic differentiation in these mice. Compared with control mice, NAC-treated recipient mice had a 10.8-fold increase in hematopoietic engraftment in the injected tibiae [85]. Recently, NAC has been used in clinical trials. A pilot study by Kong's group [89] reported an overall response of NAC treatment in 7 of 10 prolonged isolated thrombocytopenia (PT) patients without significant side effects. Furthermore, posttreatment platelet counts in the treated group of PT patients were significantly increased compared to pretreatment. Another study demonstrated that the occurrences of PGF and PT were significantly reduced, as were the ROS levels, in an EC < 0.1% patient group that received preventative NAC [90].

After NAC, resveratrol is perhaps the second most investigated antioxidant in HSC studies. The direct antioxidant effects of resveratrol as the ROS scavenger are not strong, while resveratrol can act as a gene regulator of the redox system which enhances the transcriptional activity of Nrf2 and Sirt1 [91]. Resveratrol has been used to augment *ex vivo* HSC expansion, improve BM transplantation efficiency, and—in particular—protect HSCs from IR. Human CB-HSCs are an important source for HSC transplants. Since the number of CB-HSCs is low, it is necessary to perform *in vitro* amplification before transplantation. Many small molecules including antioxidants have been tested for use in CB-HSCs *ex vivo* expansion. Schiedlmeier's group reported that the antioxidant resveratrol, together with common cytokine combinations (including stem cell factor, thrombopoietin, Fms-related tyrosine kinase 3 ligand, and interleukin-6), significantly promoted CB-HSCs *ex vivo* expansion. Moreover, resveratrol has been shown to potently support the multilineage reconstitution of human CB-HSCs in NSG (NOD/SCID/IL2r*γ*^−/−^) mice [92]. Rimmele et al. [93] reported that after mice were given resveratrol for three weeks, the number of functional HSCs significantly increased. *In vivo* experiments have shown that the function of multipotent progenitor cells is improved by resveratrol [93]. Meng's group reported that resveratrol can mitigate damage to HSCs caused by IR. Administration of resveratrol can also ameliorate acute BM symptoms and death rate and attenuate long-term HSC impairment caused by IR by reducing ROS levels [73]. Heyneanol appears to have even greater therapeutic efficacy than resveratrol and isorhapontigenin [74]. Besides NAC and resveratrol, antioxidants including curcumin [67], TMC [36], alendronate [94], vitamin A, and vitamin E have been used during BM transplantation or to treat ROS-related hematological diseases. How to select the right antioxidant for a particular patient needs to be defined from future clinical trials in the transplant patients with different antioxidants. Efficacy of a specific antioxidant for BM transplantation will largely depend on the preconditioning regimen protocol and other important factors such as disease type/stages and donor cell types/doses. The interactions of different chemotherapy drugs and antioxidants still need to be taken into consideration for choosing the right antioxidant. Furthermore, new agents targeting a specific microenvironment or cells may be also considered a combinational therapy in the future.

## 10. Concluding Remarks

In summary, primitive HSCs typically reside in the hypoxic environmental niche in the BM. Although physiological low levels of ROS are necessary to maintain functional HSCs in hemostasis, aberrantly elevated ROS levels due to intrinsic or extrinsic insults may cause significant defects and ultimately exhaustion of HSCs. In fact, high ROS levels are a known risk factor for poor BM transplantation in experimental and clinical settings. Several small molecule antioxidants have been tested to reduce ROS levels and improve the efficacy of BM transplantation, demonstrating utility in clinical stem cell transplantation.

In the future, *in situ* measurement of ROS levels and single cell metabolomics could give us a new perspective of measuring ROS variations in three-dimensional space and detecting the heterogeneity of HSPCs. Moreover, considering the largely unsuccessful result of antioxidants in cancer prevention [95], more clinical trials involving small molecule antioxidants need to be tested in application of BM transplantation. Also, the transcriptional factor Nrf2 is important in regulating redox response elements and serves as an antioxidant to reduce ROS levels. More approaches that target Nrf2 for improving efficiency of BM transplantation could be the future direction. Nonetheless, the recent Nobel-winning work on HIF-1*α* which mediated regulation in cellular oxygen sensing has some functional similarities with Nrf2 as transcriptional factors. Briefly, HIF-1*α* regulates variations in oxygen partial pressure and Nrf2 functions in redox regulation. More and more potential drugs increasing HIF-1 function [96, 97] would assist in disease treatments as well as BM transplantation. Also, it will likely fuel the future studies on the roles of ROS in hematopoiesis as well as the clinical applications of antioxidants for stem cell transplant patients.

## Abbreviations

HSCs: Hematopoietic stem cells
BM: Bone marrow
ROS: Reactive oxygen species
EPHOSS: Extra physiologic oxygen shock/stress
O_2_^−^: Superoxide anion
H_2_O_2_: Hydrogen peroxide
OH^−^: Hydroxyl ion
NOX: NADPH oxidase
SOD: Superoxide dismutase
GPX1: Glutathione peroxidase
MAPK: p38 mitogen-activated protein kinase
mTOR: Mammalian target of rapamycin
HSPCs: Hematopoietic stem and progenitor cells
CB: Cord blood
MPTP: p53-cyclophilin-mitochondrial permeability transition pore
HIF-1*α*: Hypoxia-inducible factor-1*α*
CSA: Cyclosporine A
CFCs: Colony-forming cells
NQO1: NADPH dehydrogenase quinone 1
GSR: Glutathione reductase
TXNRD1: Thioredoxin reductase 1
HO-1: Heme oxygenase 1
FTH1: Ferritin heavy chain 1
IR: Ionizing radiation
NAC: N-acetyl cysteine
G-CSF: Granulocyte colony-stimulating factor
FOXO: Forkhead O
TMC: 2-trifluoromethyl-2′-methoxychalone
GSH: Glutathione
ESCs: Embryonic stem cells
MSCs: Mesenchymal stem cells
CSCs: Cancer stem cells
LSK: Lin-Scal-1+C-Kit+
FA: Fanconi anemia
ASP: Angelica sinensis polysaccharide
DFO: Deferoxamine
DFX: Deferasirox
TBI: Total body irradiation
PGF: Poor graft function
PT: Prolonged isolated thrombocytopenia.
